# Supplementary material for: Beauty That Moves: Dance for Parkinson’s Effects on Affect, Self-Efficacy, Gait Symmetry, and Dual Task Performance
Source: Front Psychol. 2021 Feb 5;11:600440. doi: 10.3389/fpsyg.2020.600440 (PMC7892443; doi:10.3389/fpsyg.2020.600440)
Supplement: Supplementary file 1 [file Table_1.docx]

Supplementary Material

# 1 Supplementary Figures and Tables

| TUG (without cognitive task) EXAM DURATION (s) | | | | |
| --- | --- | --- | --- | --- |
| Subject ID | PRE DfPD | POST DfPD | PRE MIE | POST MIE |
| 2 | 11.49 | 13.76 | 11.49 | 12.63 |
| 3 | 11.29 | 14.28 | 12.94 | 11.95 |
| 4 | 13.14 | 14.91 | 13.20 | 13.32 |
| 5 | 12.84 | 12.01 | 11.80 | 11.90 |
| 7 | 10.97 | 10.26 | 11.10 | 11.72 |
| **mean** | **11.95** | **13.04** | **12.11** | **12.30** |
| **std** | **0.98** | **1.89** | **0.92** | **0.66** |
| TUG (with cognitive task) EXAM DURATION (s) | | | | |
| Subject ID | PRE DfPD | POST DfPD | PRE MIE | POST MIE |
| 2 | 13.14 | 12.28 | 13.68 | 13.95 |
| 3 | 13.53 | 12.73 | 13.10 | 13.59 |
| 4 | 16.13 | 17.34 | 15.40 | 15.23 |
| 5 | 15.40 | 13.94 | 14.41 | 14.26 |
| 7 | 14.80 | 12.79 | 13.29 | 13.05 |
| **mean** | **14.60** | **13.82** | **13.98** | **14.02** |
| **std** | **1.25** | **2.06** | **0.94** | **0.81** |

## Table S1: TUG Exam Duration (s) both pre- and post- Dance for PD (DfPD) and matched-intensity exercise (MIE). The upper table reports the simple TUG Exam Duration (without cognitive task) while the lower table reports the dual task TUG Exam Duration (with cognitive task). Across subjects means and standard deviations (SD) in bold.
